# The mental health impact of COVID-19 outbreak: a Nationwide Survey in Iran

**DOI:** 10.1186/s13033-021-00445-3

**Published:** 2021-02-27

**Authors:** Reza Shahriarirad, Amirhossein Erfani, Keivan Ranjbar, Amir Bazrafshan, Alireza Mirahmadizadeh

**Affiliations:** 1 Thoracic and Vascular Surgery Research Center, Shiraz University of Medical Sciences, Zand Street, Shiraz, Iran; 2 Student Research Committee, Shiraz University of Medical Sciences, Shiraz, Iran; 3 Substance Abuse Research Center, Shiraz University of Medical Sciences, Shiraz, Iran; 4 Non-Communicable Diseases Research Center, Shiraz University of Medical Sciences, Shiraz, Iran

**Keywords:** COVID-19, Depression, Anxiety, Epidemiology, Outbreak

## Abstract

**Background:**

Disease outbreak not only carries the risk of death to the public due to the infection, but it also can lead to unbearable psychological impact on the mental health of the individuals. This study aims to explore and evaluate the burden of psychological problems on the Iranian general population during the outbreak of COVID-19.

**Method:**

A cross-sectional web-based survey was conducted among the general population of Iran age 15 and above. Demographic variables, depression, and anxiety symptoms were evaluated using the Patient Health Questionnaire-9 and General Anxiety Disorder-7 questionnaires.

**Results:**

Among the 8591 participants, the mean age was 34.37 (± 11.25) years and 66.4% were female while 33.6% were male. Based on our results, 1295 (15.1%) and 1733 (20.1%) of the general population had clinically significant depressive and anxiety symptoms, respectively. Based on the demographic variables, female gender was associated with a higher risk for developing depression and anxiety symptoms, whereas getting information about the disease from medical journals and articles, being older, and being married were considered as associated protective factors. In terms of depression, being a healthcare worker was an associated risk factor. On the other hand, for anxiety, having higher education was a protective factor while a higher number of individuals in a household was considered as a risk factor.

**Conclusions:**

This study identified a major mental health problem in the Iranian population during the time of the COVID-19 outbreak. Therefore, establishing a targeted mental health support program during the time of public emergencies, such as the disease outbreak, is advised.

**Supplementary Information:**

The online version contains supplementary material available at 10.1186/s13033-021-00445-3.

## Introduction

The 2019 novel Coronavirus (COVID-19), which comes from a viral family that was thought to be rather benign before the turn of the century, has led to a public health emergency of international concern, and according to WHO is classified as pandemic [1]. The outbreak of 2019-nCoV, officially known as SARS-CoV-2 [2] was first reported in December 2019, as a cluster of acute respiratory illness in Wuhan, Hubei Province, China, and spread rapidly to other areas. Since the outbreak until 19 August 2020, the total number of confirmed cases has reached over 22,000,000 with over 780,000 deaths throughout the world [3]. Moreover, the number of infected cases is increasing and the disease is spreading to other countries in the world [4].

In Iran, the outbreak of Coronavirus was officially confirmed in Qom Province on 19 February 2020, [5, 6]. Until 19 August 2020, Iran was among the most infected country in the world with more than 340,000 cases of COVID-19 and over 19,000 deaths [3]. The absence of definite treatment, the distribution of misinformation along with the ignorance of the people and the population regarding the significance of the virus, lack of medical and hygiene supplies such as disinfectants, and masks have triggered the general population towards an increased anxiety level throughout the country [7]. Although no strict restriction has been exercised by the government for quarantining the cities, the number of travels to several provinces has been radically declined and staying indoors is widely broadcasted and advised. Furthermore, crowded places such as universities, schools, mosques, and some cultural and tourist sites have been closed until further notice. All these factors along with boredom and loneliness due to having less human interaction can pose a burden on citizens’ mental wellbeing [8, 9].

Based on the evidence demonstrating the mental health impact on individuals of previous outbreaks, such as Severe acute respiratory syndrome (SARS) [10] and Middle East Respiratory Syndrome (MERS) [11], this study aims to explore and evaluate the depressive and anxiety symptoms on the Iranian general population during the outbreak of COVID-19, to provide supporting data for introducing targeted mental health support programs for the individuals during this outbreak.

## Methods

### Study design and participants

Estimation of the sample size was done based on a study by Zhong et al. [12] by using n = Z^2^  * P (1 − P)/d2, by assuming a minimum prevalence of no knowledge 10%, confidence level = 95%, and d (margin of error) = 0.01. The calculated sample size of this study was 1382 participants, and with design effect = 2.5 reaching a sample size of nearly 3500 participants. This cross-sectional web-based survey was carried out through various social media platforms such as Instagram, WhatsApp, and Telegram. The link of the questionnaire was shared through social media in which participants could view the questions simply by clicking on the link and answer the questions. Inclusion criteria regarded all participants from 15 years and above. Also, the questionnaire was non-commercial and voluntary.

### Data collection

The questionnaire was answered by over 8500 participants anonymously from the 2nd to the 8th of March 2020. Demographic variables included age, gender, marital status, occupation (healthcare workers and non-healthcare workers), place of residence (apartment, dorm, and house), province, and the number of people living together. Also, the source of the individuals' information about the disease included social media and the internet, television, family, and friends, scientific journals and articles, as well as health care providers such as physicians and nurses were collected. Participants rated each source of information from 1 to 5 (1 as no or very low use of source and 5 as a main and dominant source). Scores ≥ 3 were categorized as a considerable source of information.

### Designed questions about the threat of COVID-19

Five questions were assigned to evaluate the individuals’ fear of the disease, and consisted of five possible answers ranging from “completely agree” to “completely disagree”. These questions are included in Table 3.

Furthermore, three questions were designed to evaluate the individuals’ perspective about the risks of the disease regarding oneself, one’s family members, and place of residence with answers varying from yes, no, and no opinion. These questions are included in Table 3.

The last question in this part was whether the participant had a history of travel within the last 30 days.

### Anxiety symptoms

In order to assess the participants’ anxiety symptoms, we used the Persian version of GAD-7 (Generalized Anxiety Disorder-7), which its validity and reliability were proved in previous studies [13]. In this questionnaire, seven questions were asked to assess the frequency of anxiety within the last 14 days, with each question having a range of scores from 0 to 3. The higher the score, the higher functional impairment due to anxiety (ranging from 0 to 21). A score of 0–4, 5–9, 10–14 and 15–21 were referred to as minimal, mild, moderate, and severe anxiety symptoms, respectively. A cutoff score ≥ 10 was defined as cases with “Anxiety” with a sensitivity of 77% and specificity of 82% [14, 15].

### Depression symptoms

In order to measure depression symptoms, the Persian version of the Patient Health Questionnaire-9 (PHQ-9) was used since its validity and reliability were proved in previous studies. It is considered as a multipurpose screening tool for monitoring, diagnosing, and determining the severity of depression [16, 17]. Nine questions were designed for evaluating the depression status of the participants with answers consisting of “Never”, “Some days”, “Most of the time” and “Always”. They were allocated scores from 0 to 4, respectively. The questionnaire consisted of two main questions, “Little interest or pleasure in activities” and “Feeling sad, depressed or hopeless”. If the participant answered “never” or “Some days” to both questions, the participant was required not to answer the remaining questions of this questionnaire and was labeled as no depression symptoms in our results. The data scale ranged from 0 to 27 and scores of under 5, 5–9, 10–14, 15–19, and 20–27 were referred to as minimal, mild, moderate, moderate to severe, and severe depression symptoms, respectively [18, 19]. A cutoff score of 10, which provides adequate sensitivity (88%) and specificity (88%), was considered for categorizing “Depression” [19]. Also, a question was asked whether answering the above-mentioned questions has affected the individual's performance in the mentioned period of time. If the answer was no, the data was ineligible and discarded from the study due to the absence of a depressive disorder.

### Statistical analysis

All the statistical analyses were performed by using Microsoft Excel 2007 (Microsoft Corp., Redmond, USA) and statistical package for social sciences (SPSS Inc., Chicago, IL, USA) version 26.0. Data are presented as mean ± standard deviation (SD) and proportions as appropriate. The Chi-square test or Fisher’s exact test was used to compare categorical data. P-values < 0.05 were considered statistically significant (two-tailed). Confidence interval (CI) of 95% and the unadjusted odds ratio were used to determine the relationship between various groups. Logistic regressions were performed on variables that were significant in bivariate analyses/Chi-square test.

## Results

In our study, 8591 filled questionnaires were received from the participants, who were from 15 to 87 years old (mean = 34.37 ± 11.25). The frequency of female participants was 5703 (66.4%) and 2888 (33.6%) were males.

As the results of our study and the scoring system from PHQ-9 show, 6680 (77.8%) of the participants had no depression symptoms, 76 (0.9%) had minimal, 540 (6.3%) had mild, 588 (6.8%) had moderate, 415 (4.8%) had moderately severe, and 292 (3.4%) cases had severe depression symptoms. Based on the cutoff mentioned in our study, 1295 (15.1%) had clinically significant depressive symptoms, while 7296 (84.9%) had no depression.

Based on GAD-7 scoring and our results, 3594 (41.8%) of participants had minimal, 3264 (38%) had mild, 1146 (13.3%) had moderate, and 587 (6.8%) cases had severe anxiety symptoms. Therefore, based on the cutoff mentioned above, 6858 (79.8%) of the participants had no anxiety, while 1733 (20.2%) had clinically significant anxiety symptoms. Table 1 demonstrates depression and anxiety symptoms among the participants, based on their demographic features.

**Table 1:** Evaluation of demographic features of the general population regarding symptoms of clinically significant depressive and anxiety

Variables	Frequency		Depressive symptoms^a^ *n* = *1295* (%)	No depression *n* = *7296* (%)	OR^b^ (95% CI)	Anxiety symptoms^a^ *n* = *1733* (%)	No anxiety *n* = *6858* (%)	OR (95% CI)
Age group (years)								
≤ 20^a^		894	199 (22.3)	695 (77.7)	–	221 (24.7)	673 (75.3)	–
21–30		2533	491 (19.4)	2042 (80.6)	1.19 (0.99–1.43)	618 (24.4)	1915 (75.6)	1.02 (0.85–1.22)
31–40		2978	414 (13.9)	2564 (86.1)	1.77 (1.47–2.14)*	595 (20)	2383 (80)	1.32 (1.10–1.57)*
41–50		1351	139 (10.3)	1212 (89.7)	2.50 (1.97–3.16)*	222 (16.4)	1129 (83.6)	1.67 (1.36–2.06)*
> 50		820	50 (6.1)	770 (93.9)	4.41 (3.18–6.11)*	74 (9)	746 (91)	3.31 (2.49–4.39)*
Sex								
Male		2888	330 (11.4)	2558 (88.6)	1.58 (1.38–1.81)*	475 (16.4)	2413 (83.6)	1.44 (1.28–1.62)*
Female^a^		5703	965 (16.9)	4738 (83.1)		1258 (22.1)	4445 (77.9)	
Marital								
Single		3544	697 (19.7)	2847 (80.3)	0.55 (0.49–0.62)*	825 (23.3)	2719 (76.7)	0.72 (0.66–0.80)*
Married^a^		5047	598 (11.8)	4449 (88.2)		908 (18)	4139 (82)	
Occupation								
Health worker^a^		1774	301 (17)	1473 (83)	1.20 (1.04–1.38)*	365 (20.6)	1409 (79.4)	1.04 (0.91–1.17)
Non-health worker		6817	994 (14.6)	5823 (85.4)		1358 (20.1)	5449 (79.9)	
Education								
Under diploma^a^		548	98 (17.9)	450 (82.1)	–	140 (25.5)	408 (74.5)	–
Diploma		1590	220 (13.8)	1370 (86.2)	1.36 (1.05–1.76)*	317 (19.9)	1273 (80.1)	1.38 (1.10–1.73)*
Academic		6453	977 (15.1)	5476 (84.9)	1.22 (0.97–1.54)	1276 (19.8)	5177 (80.2)	1.39 (1.14–1.70)*
Living place								
House/villa^a^		3238	478 (14.8)	2760 (85.2)	–	640 (19.8)	2598 (80.2)	–
Apartment		5164	782 (15.1)	4382 (84.9)	0.97 (0.86–1.10)	1058 (20.5)	4106 (79.5)	0.96 (0.86–1.07)
Dormitory		189	35 (18.5)	154 (81.5)	0.76 (0.52–1.11)	35 (18.5)	154 (81.5)	1.08 (0.74–1.58)
Number of individuals in each household								
1–4		7109	1052 (14.8)	6057 (85.2)	1.13 (0.97–1.32)	1404 (19.7)	5705 (80.3)	1.16 (1.01–1.33)*
Above 4^a^		1482	243 (16.4)	1239 (83.6)		329 (22.2)	1153 (77.8)	
History of travel								
Yes		1950	316 (16.2)	1634 (83.8)	0.89 (0.78–1.03)	385 (19.7)	1565 (80.3)	1.04 (0.91–1.18)
No^a^		6641	979 (14.7)	5662 (85.3)		1348 (20.3)	5293 (79.7)	
Source of information								
Social media/internet^a^		7119	1066 (15)	6053 (85)	–	1413 (20.1)	5688 (79.9)	-
Physicians and nurses		5794	859 (14.8)	4935 (85.2)	1.01 (0.92–1.12)	1147 (19.8)	4647 (80.2)	1.01 (0.92–1.10)
Family or friends		4513	680 (15.1)	3833 (84.9)	0.99 (0.89–1.10)	886 (19.6)	3627 (80.4)	1.02 (0.93–1.12)
Scientific articles and journals		4101	556 (13.6)	3545 (86.4)	1.12 (1.01–1.25)*	778 (19)	3323 (81)	1.06 (0.96–1.17)
Television/radio		3987	616 (15.5)	3371 (84.9)	0.96 (0.87–1.07)	819 (20.5)	3168 (79.5)	0.96 (0.87–1.06)

^a^Indicate reference level

^b^Indicator of unadjusted odds ratio, based on chi-square test

*Significant compared to the reference level; Pseudo-r-square: 2.4%

Participants were asked where they obtained the disease-related information. The findings showed that social media and the internet accounted for 82.9% of their information, family and friends 52.7%, scientific articles and journals 47.7%, physicians and nurses 67.4%, and TV and Radio 46.4%.

Based on the data from our survey, the highest depression rate was among the residence of Semnan, North Center of Iran (33.3%) and the lowest was in Lorestan, west of Iran (4.5%). Also, the highest frequency of participants with anxiety (83.9%) was seen in Alborz Province, Northern Iran, and the lowest (11.4%) in Lorestan. The Additional file 1, as provided in supplementary data, demonstrates the distribution of depression and anxiety among the participants based on their place of residence (province).

Considering the participants’ demographic features, based on chi-square test analysis, a statistically significant associations were observed between age, gender, occupation, and marital status with depression symptoms (P. value < 0.05). There was no statistically significant association between depression and the participants' education, living place (house, apartment or dorm), the number of living individuals in a household, and the history of travel (P. value = 0.071, 0.365, 0.120, and 0.113, respectively). There was a significant correlation between age group, sex, education, marital status, and the number of living individuals together with anxiety (P. value < 0.005). However, there was no significant correlation between the place of residence, the history of travel, and occupation with anxiety (P. value = 0.615, 0.608, 0.642, respectively). Nevertheless, a significant association was seen between the source of information of the participants and their level of depression and anxiety (P. value < 0.001 and 0.008, respectively).

Multiple logistic regression was performed in which the depression and anxiety were taken as a dependent while age, gender, marital status, education status, living location, history of travel, and source of information were taken as an independent factor. The findings of multiple logistic regression showed that for depression, age [β: − 0.30; OR 0.970; P. value < 0.001], male gender [β: − 0.454; OR: 0.635; P. value < 0.001], being single [β: 0.293; OR: 1.340; P. value < 0.001] were significantly associated with depression. Therefore, younger age, female gender, and being single were independent factors for depression. Also obtaining information from scientific articles and journals was significantly associated with not developing depression [for non-user/user: β: 0.231; OR: 1.260; P. value < 0.001].

Furthermore, regarding anxiety, multiple regression analysis demonstrated age [β: − 0.024; OR: 0.976; P. value < 0.001] and male gender [β: − 0.362; OR: 0.696; P. value < 0.001] were significantly associated with anxiety. Therefore, younger age and female gender were independent factors for anxiety. Moreover, obtaining information from scientific articles and journals was significantly associated with not developing anxiety [for non-user/user: β: 0.126; OR: 1.135; P. value = 0.030]. The results of multivariate regression analysis are demonstrated in Table 2.

**Table 2:** Multivariate regression of association between demographic characteristics and source of information with clinically significant depressive and anxiety symptoms during COVID-19 pandemic in Iranian population

Variables	Depression			Anxiety		
	Beta	OR (CI 95%)	P. value	Beta	OR (CI 95%)	P. value
Higher age	− 0.030	0.970 (0.963–0.977)	< 0.001	− 0.024	0.976 (0.970–0.982)	< 0.001
Gender (male/female)	− 0.454	0.635 (554–0.728)	< 0.001	− 0.362	0.696 (0.618–0.783)	< 0.001
Marital status (single/married)	0.293	1.340 (1.161–1.547)	< 0.001	0.057	1.059 (0.932–1.204)	0.379
Higher educational level	0.071	1.074 (0.967–1.192)	0.182	− 0.047	0.954 (0.872–1.045)	0.313
Living location (home/apartment or dorm)	− 0.086	0.918 (0.809–1.041)	0.180	− 0.074	0.928 (0.830–1.038)	0.192
Positive history of travel	0.072	1.075 (0.933–1.238)	0.318	− 0.051	0.950 (0.835–1.080)	0.434
Source of information (non-user/user)						
Television/radio	− 0.026	0.975 (0.864–1.100)	0.679	− 0.032	0.969 (0.870–1.079)	0.561
Social media/internet	− 0.006	1.006 (0.854–1.185)	0.946	− 0.012	0.988 (0.854–1.143)	0.869
Family or friends	0.010	0.990 (0.873–1.123)	0.878	0.070	1.072 (0.959–1.198)	0.220
Scientific articles or journals	0.231	1.260 (1.108–1.433)	< 0.001	0.126	1.135 (1.013–1.271)	0.030
Physicians and nurses	− 0.041	0.960 (0.838–1.101)	0.560	0.005	1.005 (0.891–1.135)	0.933

**Fig. 1:**
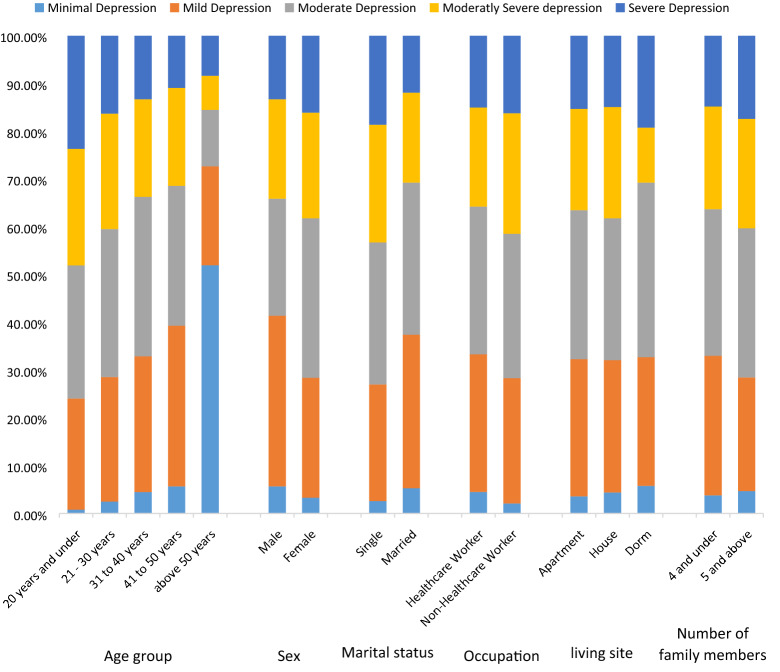
Distribution of depression symptom types among the general population based on demographic features

**Fig. 2:**
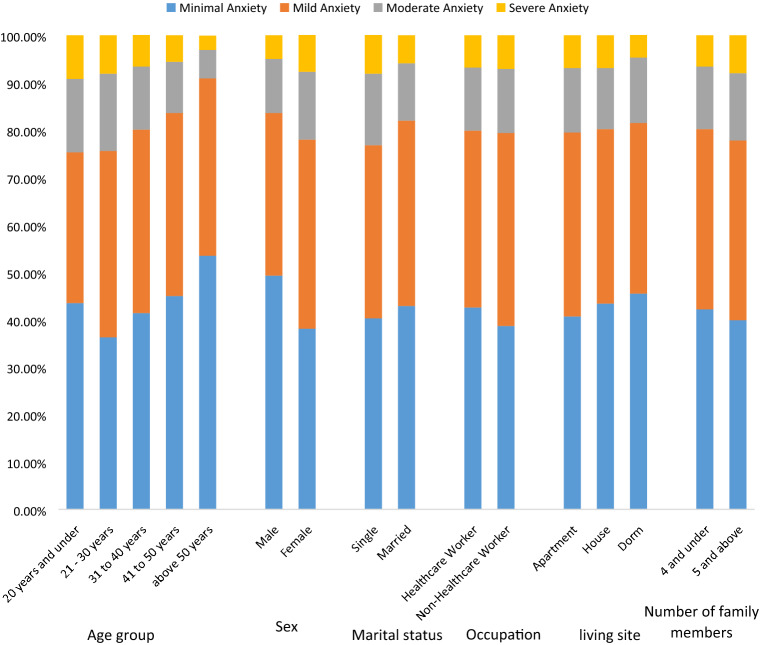
Distribution of anxiety symptom types among the general population based on demographic features

A series of questions regarding the risk and concerns about contracting the Corona virus and their presumption regarding the risk of infection were asked from the participants (Table 3). Based on our data, the individuals who were not worried or had no opinion about their own risk of contracting the disease and their family members or their living location being affected had a significantly less level of depression and anxiety compared to those who answered yes. Those who had no anxiety about contracting the disease and those who had no opinion on whether or not they are afraid of being infected had a significantly low rate of depression and anxiety (P. value < 0.001). Table 4 demonstrates the association between the attitude towards the risk of infection and depression and anxiety disorder. Also, Figs. 1 and 2 demonstrate the distribution of depression and anxiety disorder symptom types among the general population based on their demographic features, respectively.

**Table 3:** Questions regarding the risk and concerns of the general population about contracting the Corona virus

	Frequency	Completely agree	Agree	No opinion	Disagree	Completely disagree	P. value*
I’m terrified of contracting the Corona virus							
Depression							
Yes	1295	381 (29.4)	533 (41.2)	205 (15.8)	107 (8.3)	69 (5.3)	< 0.001
No	7296	1104 (15.1)	2959 (40.6)	1440 (19.7)	1094 (15)	699 (9.6)	
Anxiety							
Yes	1733	656 (37.9)	641 (37)	223 (12.9)	120 (6.9)	93 (5.4)	< 0.001
No	6858	829 (12.1)	2851 (41.6)	1422 (20.7)	1081 (15.8)	675 (9.8)	
Due to the anxiety of contracting the Corona virus, I avoid going to public places							
Depression							
Yes	1295	529 (40.8)	487 (37.6)	94 (7.3)	123 (9.5)	62 (4.8)	< 0.001
No	7296	2116 (29)	3104 (42.5)	569 (7.8)	1002 (13.7)	505 (6.9)	
Anxiety							
Yes	1733	760 (43.9)	627 (36.2)	114 (6.6)	146 (8.4)	86 (5)	< 0.001
No	6858	1885 (27.5)	2964 (43.2)	549 (8)	979 (14.3)	481 (7)	
Because I am afraid of being infected by the Corona virus, I avoid shaking hands and touching suspicious surfaces							
Depression							
Yes	1295	723 (55.8)	468 (36.1)	46 (3.6)	32 (2.5)	26 (2)	< 0.001
No	7296	3505 (48)	3122 (42.8)	227 (3.1)	244 (3.3)	198 (2.7)	
Anxiety							
Yes	1733	1036 (59.8)	566 (32.7)	63 (3.6)	34 (2)	34 (2)	< 0.001
No	6858	3192 (46.5)	3024 (44.1)	210 (3.1)	242 (3.5)	190 (2.8)	
I’m petrified that if I contract the Corona virus, I will die							
Depression							
Yes	1295	307 (23.7)	355 (27.4)	250 (19.3)	227 (17.5)	156 (12)	< 0.001
No	7296	724 (9.9)	1566 (21.5)	1606 (22.0)	2003 (27.5)	1397 (19.1)	
Anxiety							
Yes	1733	483 (27.9)	497 (28.7)	340 (19.6)	232 (13.4)	181 (10.4)	< 0.001
No	6858	548 (8)	1424 (20.8)	1516 (22.1)	1998 (29.1)	1372 (20)	
I’m anxious about the chances of me getting infected with Corona virus to be high							
Depression Yes	1295	134 (10.3)	288 (22.2)	423 (32.7)	340 (26.3)	110 (8.5)	< 0.001
No	7296	211 (2.9)	947 (13)	2127 (29.2)	2625 (36)	1386 (19)	
Anxiety Yes	1733	182 (10.5)	402 (23.2)	622 (35.9)	393 (22.7)	134 (7.7)	< 0.001
No	6858	163 (2.4)	833 (12.1)	1928 (28.1)	2572 (37.5)	1362 (19.9)	

Presumption of risk of infection due to Corona virus	Frequency	Agree	No opinion	Disagree	P. value
Are you worried about contracting the Corona virus?					
Depression (%)	1295	840 (64.9)	209 (16.1)	246 (19)	< 0.001
No depression (%)	7296	3691 (50.6)	2307 (31.6)	1298 (17.8)	
Anxiety	1733	1246 (71.9)	211 (12.2)	276 (15.9)	< 0.001
No anxiety	6858	3285 (38.2)	1296 (18.9)	2277 (33.2)	
Are you anxious about your family members contracting the Corona virus?					
Depression	1295	1189 (91.8)	63 (4.9)	43 (3.3)	< 0.001
No depression	7296	6234 (85.4)	671 (9.2)	391 (5.4)	
Anxiety	1733	1630 (94.1)	45 (2.6)	58 (3.3)	< 0.001
No anxiety	6858	5793 (84.5)	389 (5.7)	676 (9.9)	
Are you worried about your living place getting contaminated with the Corona virus?					
Depression	1295	1105 (85.3)	86 (6.6)	104 (8)	< 0.001
No depression	7296	5436 (74.5)	659 (9)	1201 (16.5)	
Anxiety	1733	1547 (89.3)	74 (4.3)	112 (6.5)	< 0.001
No anxiety	6858	4994 (72.8)	671 (9.8)	1193 (17.4)	

^a^Chi-square test

**Table 4:** Distribution of clinically significant depressive and anxiety symptoms among the Iranian general population based on their presumption regarding the risk of infection

	Frequency	Depressive symptoms (%)	No depression (%)	OR^b^ (95% CI)	Anxiety symptoms (%)	No anxiety (%)	OR (95% CI)
Are you worried about contracting the Corona virus?							
Yes^a^	4531	840 (64.9)	3691 (50.6)	–	1246 (71.9)	3285 (47.9)	–
No	2553	246 (19)	2307 (31.6)	2.13 (1.84–2.48)*	276 (15.9)	2277 (33.2)	3.13 (2.72–3.60)*
No opinion	1507	209 (16.1)	1298 (17.8)	1.41 (1.20–1.67)*	211 (12.2)	1296 (18.9)	2.33 (1.99–2.73)*
Are you anxious about your family members contracting the Corona virus?							
Yes^a^	7423	1189 (91.8)	6234 (85.4)	–	1630 (94.1)	5793 (84.5)	–
No	734	63 (4.9)	671 (9.2)	2.03 (1.56–2.65)*	58 (3.3)	676 (9.9)	3.28 (2.49–4.31)*
No opinion	434	43 (3.3)	391 (5.4)	1.73 (1.26–2.39)*	45 (2.6)	389 (5.7)	2.43 (1.78–3.33)*
Are you worried about your living place getting contaminated with the Corona virus?							
Yes^a^	6541	1105 (85.3)	5436 (74.5)	–	1547 (89.3)	4994 (72.8)	–
No	1305	104 (8)	1201 (16.5)	2.35 (1.90–2.90)*	112 (6.5)	1193 (17.4)	3.30 (2.70–4.04)*
No opinion	745	86 (6.6)	659 (9)	1.56 (1.23–1.97)*	74 (4.3)	671 (9.8)	2.81 (2.20–3.60)*

^a^Indicate reference level

^b^Indicator of unadjusted odds ratio based on Chi-square test

*Significantly compared to the reference level

## Discussion

Similar to other studies in the literature, several studies about the impact of public health emergencies such as SARS [20] and Ebola [21, 22] outbreaks on the public mental health problems were presented. Currently, Iran is the 4th most infected country with COVID-19 in the world after China, Italy, and Spain. Rapidly increasing numbers of cases and deaths in the past weeks, lack of medical facilities and protective tools and receiving contradictory news about the nature of the disease have caused fear, distress, and panic among the general population of Iran. On the other hand, health care workers have faced serious challenges due to the inadequacy of protective equipment and the stretching of hospitals to the breaking point due to the rapidly increasing number of patients seeking medical treatment.

Our cross-sectional survey demonstrated that 15% and 20% of participants reported indicators of depression and anxiety, respectively. Being female, young, and single are considered as associating factors for depression and anxiety. Furthermore, the higher number of individuals in a household was also an additional related factor for anxiety and being a health care worker as an associated factor for depression. Our data also showed that receiving information about the disease from scientific articles and journals was considered as a related protective factor from depression whereas having higher education was considered a related protective factor for both anxiety and depression.

Several studies have reported the prevalence of depression and anxiety in the Iranian population in the past. According to a national survey of the mental health of Iranians in 2011, the levels of depression and anxiety were reported 12.7% and 15.6%, respectively [23]. In a survey on the adult population of Iran, Noorbala et al. reported the depression rate of 10.4% in 2015 [24]. The already existing level of anxiety and depression would most likely rise among the general population of Iran during the COVID-19 outbreak. Similar studies during the COVID-19 outbreak in China reported higher rates of anxiety and depression which ranged from 34 to 48% and 18 to 22%, respectively [25, 26]. Compared to the Iranian population, these higher rates of anxiety and depression could be due to the strict quarantine by the government of China and the higher rate of infected cases and deaths, or because of using different methods and cut off points for the questionnaires. Previous studies have shown quarantining to be a predictive factor for developing depression up to 3 years post-outbreak due to risk factors such as inadequate supplies and financial loss [27, 28].

Our results showed that females and younger people are at a higher risk of developing anxiety and depression compared to males and older individuals. This was confirmed by what Huang et al. reported in the outbreak of COVID-19 in China [25]. A WHO based survey reported that the rate of depression decreases as people get older, even though it is accompanied by other comorbid diseases [29]. Additionally, studies on the Iranian population stated that females are at higher risk for psychiatric diseases [30, 31].

In a study by Gao et al. [26], married individuals were more prone to anxiety. This is in contrast to our results that demonstrated marriage to be a related protective factor for both anxiety and depression, which is also supported by other studies [32, 33]. This might be caused by marriage as an element for an increased human to human interaction which can subsequently reduce the risk of mental health problems. Our study also showed that crowded households (above 4 individuals) had higher levels of anxiety and those who lived in dormitories had higher levels of depression. In a study that was done during the SARS outbreak in 2007, Su et al. stated that those who had been diagnosed with depression had poor neighborhood relationships [34]. These results can be supported by the fact that although factors such as close human to human contact in small families and marriage can protect the person against mental disorders, more crowded households and environments can increase the chance of infection by the virus due to increased contact, and result in augmented anxiety and depression during outbreaks.

Our study demonstrated that individuals with lower levels of education had a risk of developing depression and anxiety. This might be due to the fact that people with higher education have better knowledge about the virus and are able to take protective measures against it and, as a result, have lower levels of depression and anxiety. This is supported by Gao et al. who presented in their study that higher educational level results in lower levels of anxiety and depression [26]. Additionally, those who received information about the disease from scientific articles and journals, rather than other sources such as social media, had a lower rate of depression and anxiety. It can be inferred that evidence-based information from scientific articles can reduce depression and anxiety by providing the reader with trustworthy information. Similar studies also reported that people who used mass media as their source of information had higher rates of depression due to “infodemic” [26].

Based on our data, the individuals who did not worry about the disease infecting themselves, their family members, or infecting their living place had a significantly less level of depression and anxiety. So, the virus jeopardizes the individual both physically and mentally. The highest concern among people with anxiety and depression was the infection of a family member. That is why most of them agreed to avoid contact with suspicious or infected surfaces and individuals. These results show that being worried about the close ones at the time of outbreak can have a burden on the mental health of individuals in society.

It is worth mentioning that based on our results, individuals believed that compared to others, they are less at the risk of contracting the virus. Similar results were also reported by Klein et al. in China [35]. Individuals, when addressing risks that familiar and based on volitional control, tend to have a more optimistic approach towards the disease while they act more pessimistic towards risks that are less under their control and mainly affected by others [35–38]. Both cognitive and emotional matters including fear and worry have a significant attitude on managing health threats. In other words, since the individual is in control of avoiding the contraction of the disease, he or she is less anxious; since they are both worried about their dear ones and are not in control of their protective measures. This understanding and emotional bond makes the individual struggle to manage their health threats.

It seems that numerous factors are affecting the mental status of the Iranian population alongside the pandemic. Among these one can name the firm sanctions against Iran. Although sanctions against Iran have been in place for the last 40 years since the Islamic Revolution and have covered nearly all sectors, such as insurance, banks, energy, commerce, and transport [39], The COVID-19 pandemic in Iran coincides with the country’s ever-highest politically driven sanctions and amid a national economic downturn in which sharp spikes in the price of medication have infected over 6 million patients with complicated and chronic diseases [40]. Even before COVID-19, the healthcare system in Iran had felt the burden of the sanctions [41]. Their impact is now severe in that they restrict the capacity of the government to raise funds or import essential goods [42, 43]. This issue has caused a dramatic social concern leading people to excessively purchasing and hoarding medical supplies, resulting in a shortage in other areas. Also, the pandemic had a significant impact on the healthcare workers with a high mortality rate [44] Every member of the medical staff who died from the disease was declared a martyr and a national hero; this reflected the moral dimension of the issue and helped strengthen efforts to combat the disease and win public support for health workers.

Among the other factors is the army which adopted a wartime attitude. All religious ceremonies, including religious congregations and masses and Friday prayers, were closed along with universities and schools, entertainment centers, theatres, cinemas, and sporting events and gymnasiums; car and real estate transactions decreased; hotels and accommodation centers received nearly zero guests [45]. This causes the public to be deprived of entertainment centers, as well as impacting the religious population worshiping habits, as Iran is amongst the religious countries in the Middle East, which one cannot deny the effects of these factors on the individuals’ mental health.

Of course, the COVID-19 pandemic in Iran also led to good events, one of which was greater popular solidarity. There was not one report in Iran about the invasion or looting of shops. People also disinfected passages and ATMs. Some landlords forgave the rental payment, and household workshops were opened to produce masks. Around the same period, people formed an intimate bond with the health care professionals, and several retailers also offered health care staff discounts. Threats changed into opportunities. Pollution decreased in some cities and the Islamic Republic of Iran Broadcasting motivated people to read books and watch movies. The release of several inmates was also good news. However, on the other hand, some disasters took place, such as misinformation on social media regarding the impact of consuming alcohol on disease prevention—and since alcoholic drinks are illegal in Iran—caused numerous deaths and methanol poisoning in different cities, especially in southern Iran [46]. As hospitals faced a lack of ICU beds for COVID-19 patients, alcohol poisoning doubled the burden on healthcare and medical systems. Also, on March 28, inmates in several prisons were distressed enough that they clashed with guards, set fire to prisons, and somehow escaped. Jahanshahi et al. suggest that adults in Iran suffer more distress than adults in China, with levels of distress predicted by various factors [47]. Our survey in Iran was conducted during the early days of the reported outbreak, however, it is still hard to evaluate the effect of the policies exercised by the government and people on the populations’ mental health status, however, one cannot deny that special attention should be given regarding safeguarding the populations mental alongside their physical health to avoid long-lasting and even permanent consequences.

As the final point, the results of this study shed light on the unseen burden of COVID-19 outbreak on the mental health of the general population of Iran. Although locking down the cities can have a good effect on controlling the spread of the disease, it can also lead to more serious mental health problems in that area. Health authorities should be aware of this burden and be prepared to take immediate action whenever needed, particularly targeting groups at higher risks such as younger and female individuals. Furthermore, the implantation of preventive measures, especially for these risk groups, could be beneficial for further similar situations and public health emergencies [48].

One of the strengths of our study was the significant number of participants in the early days of the outbreak. On the other hand, this study has several limitations too. Firstly, the study was cross-sectional which makes it difficult to precisely explain causal relationships. Therefore, further longitudinal studies are essential to be conducted in the future. Secondly, since SARS-CoV-2 can be transmitted via droplets or close contacts, a web-based approach was adapted for this study. However, there are several selection biases such as illiteracy and the absence of internet access and an over-representation of females and higher-educated individuals. Since educational attainment and occupation are frequently considered as proxy measures of socioeconomic status [49], our results can only be comprehensive to relatively high socioeconomic status, particularly female Iranians. Moreover, no data regarding the previous mental health status of individuals were obtained nor any pre-pandemic mental health data assessments were available for comparison.

## Conclusion

In conclusion, this study identified a major mental health burden on public health during the time of the COVID-19 outbreak in Iran. Females and younger adults are at higher risk of developing mental health problems compared to those who have higher education and those who obtain their information about the disease from scientific articles and journals. Therefore, establishing a targeted mental health support program, surveillance, and monitoring of consequences of psychological problems during the time of public emergencies such as disease outbreaks is advised.

## Supplementary Information

**Additional file 1: **Distribution of depression and general anxiety among the Iranian general population based on province.

## Data Availability

Data are attached as supplementary materials, and information related to the study is in the manuscript. Please contact the corresponding author for any further data.
