# Supplementary material for: The mental health impact of COVID-19 outbreak: a Nationwide Survey in Iran
Source: Int J Ment Health Syst. 2021 Feb 27;15:19. doi: 10.1186/s13033-021-00445-3 (PMC7913044; doi:10.1186/s13033-021-00445-3)
Supplement: Supplementary file 1 — Additional file 1: Distribution of depression and general anxiety among the Iranian general population based on province. [file 13033_2021_445_MOESM1_ESM.docx]

Additional file 1: Table S1 Distribution of depression and general anxiety among the Iranian general population based on province

| **Province** | Frequency | Depression (%) | No Depression (%) |  | Anxiety (%) | No Anxiety (%) |
| --- | --- | --- | --- | --- | --- | --- |
| Fars | 4416 | 635 (14.4) | 3781 (85.6) |  | 877 (19.9) | 3539 (80.1) |
| Tehran | 1110 | 192 (17.3) | 918 (82.7) |  | 246 (22.2) | 864 (77.8) |
| Bushehr | 432 | 53 (12.3) | 379 (87.7) |  | 85 (19.7) | 347 (80.3) |
| Khuzestan | 407 | 59 (4.6) | 348 (85.5) |  | 71 (17.4) | 336 (82.6) |
| Isfahan | 359 | 62 (17.3) | 294 (82.7) |  | 86 (24) | 273 (76) |
| Hormozgan | 219 | 26 (11.9) | 193 (88.1) |  | 40 (18.3) | 179 (81.7) |
| Gilan | 205 | 36 (17.6) | 169 (82.4) |  | 38 (18.5) | 167 (81.5) |
| Alborz | 174 | 21 (12.1) | 153 (87.9) |  | 28 (83.9) | 146 (16.1) |
| Khorasan Razavi | 170 | 28 (16.5) | 142 (83.5) |  | 35 (20.6) | 135 (79.4) |
| Kerman | 151 | 24 (15.9) | 127 (84.1) |  | 33 (21.9) | 118 (78.1) |
| Mazandaran | 104 | 17 (16.3) | 87 (83.7) |  | 16 (15.4) | 88 (84.6) |
| Kohgiluyeh and Boyer Ahmad | 96 | 15 (15.6) | 81 (84.4) |  | 15 (15.6) | 81 (84.4) |
| Yazd | 77 | 11 (14.3) | 66 (85.7) |  | 16 (20.8) | 61 (79.2) |
| Other locations | 66 | 7 (10.6) | 59 (89.4) |  | 12 (18.2) | 54 (81.8) |
| East Azerbaijan | 62 | 10 (16.1) | 52 (83.9) |  | 17 (27.4) | 45 (72.6) |
| Kermanshah | 52 | 9 (17.3) | 43 (82.7) |  | 10 (19.2) | 42 (80.8) |
| West Azerbaijan | 49 | 8 (16.3) | 41 (83.7) |  | 10 (20.4) | 39 (79.6) |
| Golestan | 48 | 9 (18.8) | 39 (81.3) |  | 8 (16.7) | 40 (83.3) |
| Lorestan | 44 | 2 (4.5) | 42 (95.5) |  | 5 (11.4) | 39 (88.6) |
| Qom | 42 | 11 (26.2) | 31 (73.8) |  | 9 (21.4) | 33 (78.6) |
| Sistan and Baluchestan | 40 | 7 (17.5) | 33 (82.5) |  | 10 (25) | 30 (75) |
| Hamedan | 36 | 8 (22.2) | 28 (77.8) |  | 8 (22.2) | 28 (77.8) |
| Markazi | 36 | 6 (16.7) | 30 (83.3) |  | 6 (16.7) | 30 (83.3) |
| Chaharmahal Bakhtiari | 28 | 6 (21.4) | 22 (78.6) |  | 4 (14.3) | 24 (85.7) |
| Ghazvin | 28 | 3 (10.7) | 25 (89.3) |  | 8 (28.6) | 20 (71.4) |
| Kordestan | 25 | 8 (32) | 17 (68) |  | 10 (40) | 15 (60) |
| Zanjan | 22 | 5 (22.7) | 17 (77.3) |  | 6 (27.3) | 16 (72.7) |
| Ardebil | 22 | 3 (13.6) | 19 (86.4) |  | 9 (40.9) | 13 (59.1) |
| Semnan | 21 | 7 (33.3) | 13 (66.7) |  | 4 (19) | 17 (81) |
| Ilam | 18 | 1 (5.6) | 17 (94.4) |  | 3 (16.7) | 15 (83.3) |
| Northern Khorasan | 16 | 2 (12.5) | 14 (87.5) |  | 4 (25) | 12 (75) |
| Southern Khorasan | 16 | 4 (25) | 12 (75) |  | 4 (25) | 12 (75) |
